# Maternal Mid-Gestation Cytokine Dysregulation in Mothers of Children with Autism Spectrum Disorder

**DOI:** 10.1007/s10803-021-05271-7

**Published:** 2021-09-09

**Authors:** S. Casey, M. Carter, A. M. Looney, V. Livingstone, G. Moloney, G. W. O’Keeffe, R. S. Taylor, L. C. Kenny, F. P. McCarthy, L. M. E. McCowan, J. M. D. Thompson, D. M. Murray, the SCOPE Consortium

**Affiliations:** 1 Irish Centre for Foetal and Neonatal Translational Research, University College Cork, Cork, Ireland; 2 Department of Paediatrics and Child Health, University College Cork, Cork, Ireland; 3 Department of Anatomy and Neuroscience, University College Cork, Room 2.33, Western Gateway Building, Western Road, Cork, Ireland; 4 Department of Obstetrics and Gynaecology, University of Auckland, Auckland, New Zealand; 5 Department of Paediatrics, Child and Youth Health, University of Auckland, Auckland, New Zealand

**Keywords:** IL-17A, Autism spectrum disorder, Cytokine, Inflammation, Maternal immune activation

## Abstract

Autism spectrum disorder (ASD) is a developmental disorder characterised by deficits in social interactions and communication, with stereotypical and repetitive behaviours. Recent evidence suggests that maternal immune dysregulation may predispose offspring to ASD. Independent samples t-tests revealed downregulation of IL-17A concentrations in cases, when compared to controls, at both 15 weeks (p = 0.02), and 20 weeks (p = 0.02), which persisted at 20 weeks following adjustment for confounding variables. This adds to the growing body of evidence that maternal immune regulation may play a role in foetal neurodevelopment.

## Introduction

ASD is an intricate continuum of neurodevelopmental disorders all of which have an onset in early childhood. These disorders are characterised by impairments in social interaction and communication, and the presence of restricted, ritualistic or repetitive interests, behaviours and activities (Birtwell, 2016; Magiati et al., 2016). To meet the diagnostic criteria, symptoms must have been present during the early developmental period, and must cause significant functional impairments (social or occupational) of varying severities (American Physchiatric Association, 2013). It reportedly affects approximately 1.5% of the population in the developed world (Lyall et al., 2017). Although deficits can be present from infancy, diagnosis is often delayed. Classic Autism is typically formally diagnosed at an average of 5.6 years (standard deviation (SD) 4.1), and Asperger’s at an average of 9.9 years (standard deviation (SD) 5.3) (Crane et al., 2015). An early, accessible biomarker which could aid early detection and intervention (Boyd et al., 2012) would be a significant step forward in the care of these children.

There is growing evidence that disturbance of inflammatory and immune responses may be a significant contributing factor behind the pathophysiology of many psychiatric disorders (Kim et al., 2007; Masi et al., 2015, 2017; Müller et al., 2015). Alterations of immune cell expression have been documented repeatedly in ASD affected children and adults as well as animals with an ASD-like phenotype (Akintunde et al., 2015; Ashwood et al., 2011; Fernández de Cossío et al., 2017), and maternal viral or bacterial infections have been found to be significantly associated with ASD in offspring (Malkova et al., 2012). Maternal immune activation (MIA) is believed to disrupt the delicate processes underlying neuronal development, increasing the risk of disordered neurodevelopment (Deverman & Patterson, 2009; Garay & McAllister, 2010).

MIA may typically be modelled in animals using lipopolysaccharide (LPS), Polyinosinic:polycytidylic acid (Poly(I:C)), or valproic acid. MIA in rodents results in a wide array of enduring ASD-like behavioural alterations in offspring. Neurodevelopment of the rodent brain is said to be equivalent to that noted in human mid-gestational neurodevelopment between gestational days 10–20 (Patten et al., 2014). Inflammatory insults during this time have resulted in reductions in social approach and reciprocal social behaviour, increases in repetitive and stereotypical behaviours, typically measured using a marble burying test, abnormal prepulse inhibition and ultrasonic vocalisations, impaired learning and memory, measured using a variety of maze tests, and reduced novel object recognition (Boksa, 2010; Careaga et al., 2017). Few large models of MIA induced ASD exist, though non-human primate models are more common than others, and extend findings in rodent models. A mid-gestation viral challenge in the rhesus monkey may manifest as repetitive behaviours, decreased affiliative vocalisations, inappropriate social interactions with novel animals, and impaired social attention (Bauman et al., 2014; Machado et al., 2015).

Human epidemiological studies have shown that immune disorders and mid-trimester viral illnesses which lead to a pro-inflammatory state in mothers during pregnancy, are associated with increased ASD, schizophrenia and bipolar disorder risk in offspring (Atladottir et al., 2010; Chess, 1977; Conway & Brown, 2019; Jiang et al., 2016). In 1977, Chess noted ASD rates of 8–13% in offspring of United States (US) mothers who were infected in the 1964 Rubella outbreak (Chess, 1977). More recently, Maher et al. linked preeclampsia to increased ASD risk (Maher et al., 2020).

Midgestation in particular appears to be an important neurodevelopmental period. Some of the key processes occurring during this period include the development of the hippocampus, cortical plate, the longitudinal fissure, sulci and gyri, cerebellum, superior and inferior colliculi, primary visual, motor and sensory cortices, the cerebrospinal tract, as well as spinal cord myelination, as well as neurogenesis. The brain also significantly increases in size between gestational weeks 13 and 21 (Huang et al., 2009; Joseph, 2000; Prayer et al., 2006; Stiles & Jernigan, 2010). Insults during this time have been found to result in neurodevelopmental and psychiatric disorders in both humans and animals (Buss et al., 2010; Haddad et al., 2020; Wolff & Bilkey, 2008).

Very few clinical studies have examined the cytokine profiles of mothers who go on to have a child with ASD. A retrospective 2017 study reported elevated levels of several circulating cytokines and chemokines in mid-gestational mothers who progressed to bear a child affected by ASD. This study was able to examine children with an early diagnosis of ASD, with and without intellectual disability. These included granulocyte–macrophage colony-stimulating factor (GM-CSF), IL-1α, IL-6, interferon-γ (IFN-γ), IL-8 and monocyte chemoattractant protein-1 (MCP-1) (Jones et al., 2017). An earlier study performed by Goines et al. showed dysregulation in a number of serum cytokines including IFN-γ, IL-4, IL-5 and IL-10 at a single time point between 15 and 19 weeks’ gestation (Goines et al., 2011). Elevated MCP-1 has also been observed in amniotic fluid samples of ASD infants (Abdallah et al., 2012). Brown et al. identified increased levels of the inflammatory marker C-reactive protein (CRP) in prospectively collected maternal serum samples during early pregnancy (Brown et al., 2014). In recent times more conditions with a pro-inflammatory milieu, such as obesity, psychosocial stress and pre-eclampsia have also been reported to increase the risk of ASD (Curran et al., 2018; Knuesel et al., 2014; Maher et al., 2020). Thus, MIA and cytokine dysregulation during pregnancy seems to play a role in the pathogenesis of the ASD phenotype.

In the present study, we wished to examine the mid-gestational cytokine profiles in mothers of children with a subsequent ASD diagnosis examined at two mid-gestation time points (15 and 20 weeks) across two sites of a large multi-centre pregnancy study with the aim of identifying a gestational ASD biomarker which may aid in the timely treatment and management of the disorder.

## Methods

### Study Population

Maternal-child dyads were recruited to the population-based SCOPE study (www.scopestudy.net). This study used a cohort from the two SCOPE centres from which paediatric follow-up was completed. These sites were Cork, Ireland (Cork ECM5 (10) 05/02/08) and Auckland, New Zealand (SCOPE-NZ) (AKX/02/00/364). In Cork, children had detailed follow-up from birth to 5 years through the Cork BASELINE Birth Cohort Study. All children who scored below the “cutoff value” in the Ages and Stages Questionnaire (suggestive of abnormal development) were referred for paediatric assessment. Those with suspected ASD at 2 or 5 years were referred to early intervention services for full ASD assessment. Further follow-up was completed after Early Intervention Services (EIS) assessment to confirm diagnosis of ASD. Diagnosis was considered to be confirmed if made by a professional (EIS or child psychiatrist). In Auckland, telephone follow-up using standardised questionnaires was carried out as part of the Children of SCOPE study at 6 years and ASD diagnosis was by parent report. Cases from both sites were enrolled to the cohort.

Inclusion criteria for enrolment were:

- Biobanked maternal antenatal serum samples
- Developmental follow-up completed for the child at 5 or 6 years of age (site dependant)
- Cases had a diagnosis of ASD according to the local selection criteria
- Controls had no underlying medical or developmental conditions

**Fig. 1:**
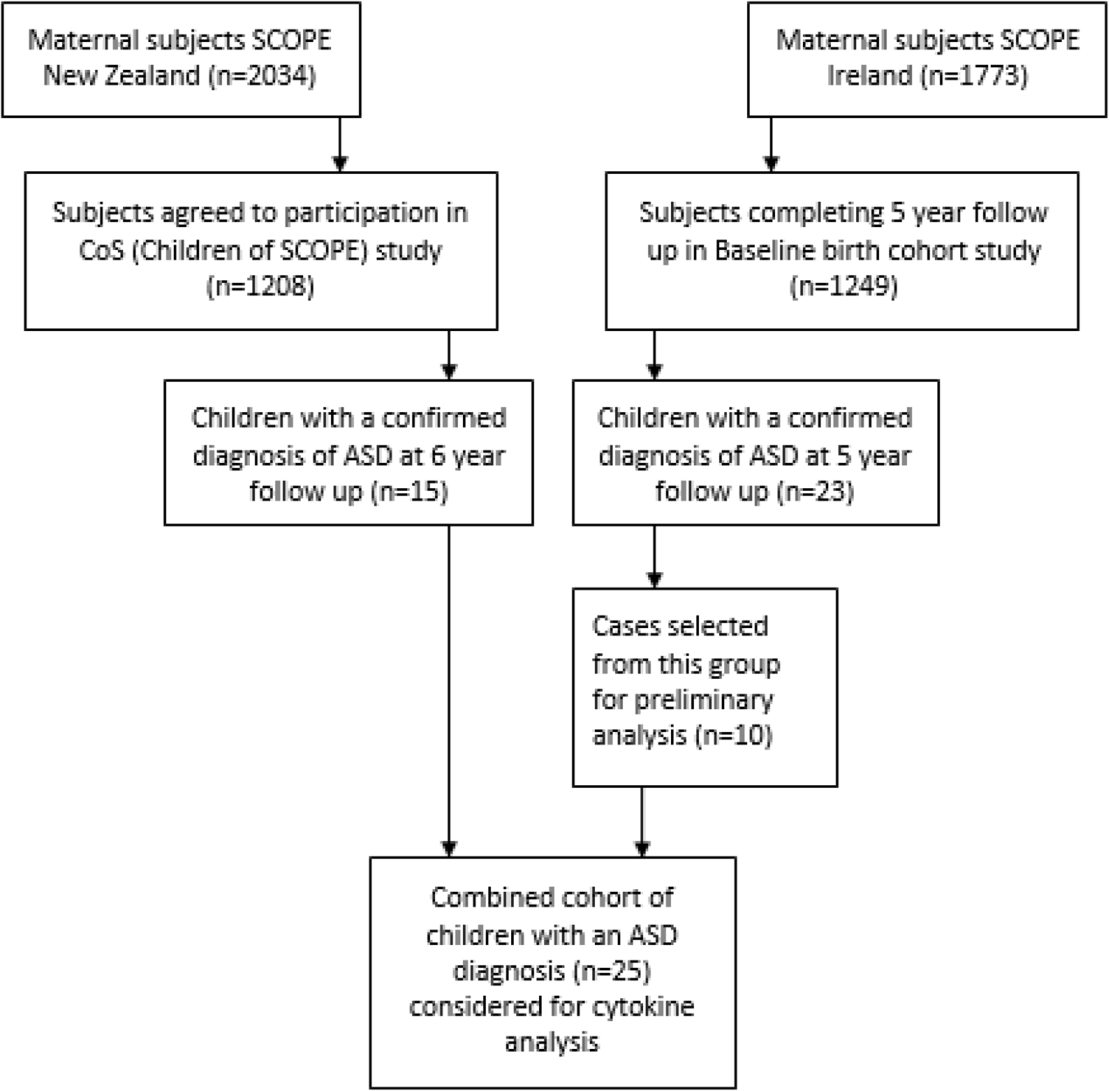
Patient recruitment flow chart outlining participant enrolment and and follow up across both sites

SCOPE-IRELAND and the Cork BASELINE Birth Cohort study was carried out with local ethical approval from the Cork Research Ethics Committee (Cork ECM5 (10) 05/02/08). Full written informed consent was obtained in all cases. SCOPE-NZ and the Children of SCOPE study was carried out with ethical approval gained from local ethics committees (New Zealand Health and Disability Ethics Committees (AKX/02/00/364 and NTX/10/10/106) and all women provided written informed consent. A patient recruitment flowchart is outlined in Fig. 1.

### Demographic Variables

Demographic and relevant clinical data regarding the participants is presented in Table 3. ‘Age, maternal’ represents maternal age in years at the time they were approached to participate in the study whilst pregnant. ‘Birthweight, g’ is the infant’s birthweight in grams. ‘SEI’ stands for Socioeconomic Index and this variable was calculated using the New Zealand Socioeconomic Index guide. The same index was used across both locations, and Cork participants were scored based on the same criteria as their New Zealand counterparts (Galbraith et al., 1996). Perceived Stress Scores (PSS) are based on the ten question PSS questionnaire (Cohen et al., 1994). An individual’s scores on the PSS can range from 0 to 40 with higher scores indicating higher perceived stress. Low stress scores range from 0 to 13, moderate stress scores range from 14 to 26, and high stress scores range from 27 to 40. Gestational age at delivery is presented in weeks, and APGAR (appearance, pulse, grimace, activity and respiration) scores are presented as being < 7 or ≥ 7 at both 1 and 5 min of age. Household income represents the combined household income and is quoted in New Zealand Dollars ($) and Euros (€). Body Mass index (BMI) is categorised using the World Health Organisation (WHO) criteria and is measured in kilograms per metre squared. Underweight and normal BMI categories are considered together as are overweight and obese categories. Folate intake was categorised as yes or no for: (i) any supplemental folate in the preconceptual period and (ii) at the 15 week visit.

### Biofluid Collection

Serum samples were obtained from mothers recruited to the SCOPE-NZ and SCOPE-Cork studies at both 15 and 20 weeks gestation within the greater Auckland area, New Zealand and Cork Univeristy Maternity Hospital, Cork, Ireland. Biobank specimens were archived at − 80 °C until required. Maternal mid-pregnancy specimens from 15 and 20 weeks were retrieved from the multi-centre SCOPE study sites with ongoing paediatric follow-up. Identical protocols for collection, processing and storage of samples were followed at both sites.

Venepunture was performed by SCOPE study specific research midwives at each of the sites in accordance with best practice guidance (SCOPE Consortium standard operating procedures (SOP)). Maternal specimens were collected in serum separator tubes (Becton–Dickinson Frankin Lakes, New Jersey) and immediately placed on ice and transported to the laboratory. Before proceeding to centrifugation, serum samples were stored at 4 °C for 30 min from time of collection to allow clot formation. Presence of the clot was confirmed visually, and samples were then centrigued at 2400×*g* for 10 min at 4 °C. Serum samples were transferred into ice cold 5 mL sterile polypropylene tubes (VWR, Radnor, Pennsylvania) via sterile Pasteur pipettes. The samples were centrifuged again at 3000×*g* for 10 min at 4 °C. Sera were then aliquoted to red capped, barcode-labelled cryovials (VWR) in volumes of 250 µl. Aliquots were logged in the SCOPE database (MedSciNet), and stored at − 80 °C within four hours of collection(Kenny et al., 2014). For transport of NZ serum samples to Cork, Ireland: the maternal specimens were packed on dry ice and shipped directly to the SCOPE Ireland biobank repository, where they were stored at − 80 °C until their use in cytokine and chemokine profiling.

### Cytokine Analysis

Serologic concentrations (pg/ml) of eight cytokines, chemokines and proinflammatory proteins were investigated at 15 and 20 weeks gestation using the Mesoscale Discovery V-plex cytokine, chemokine and proinflammatory electrochemiluminescent assays (Meso Scale Diagnostics, Rockville, Maryland). Cytokines were chosen for further examination based on evidence of dysregulated expression in preclinical models (Choi et al., 2016; Pineda et al., 2013; Pratt et al., 2013; Smith et al., 2007) and autistic patients (Ahmad et al., 2019; Ashwood et al., 2011; Masi et al., 2015; Patterson, 2011).

IL-16 and IL-17A were examined using the V-plex multiplex Cytokine Panel 1 kit (KD15050D). Eotaxin and MCP-1 were examined using the V-plex multiplex Chemokine Panel 1 kit (K15047D). IFN-γ, IL-1β, IL-6 and IL-8 were examined using the V-plex multiplex Proinflammatory Panel 1 kit (K15049D). All standards and samples were run in duplicate.

All plates were prepared according to manufacturer’s instructions and analysed on the Meso QuickPlex SQ 120. Results were generated as calculated concentration means on the Mesoscale (MSD) Discovery Workbench 4.0 assay analysis software. Calibration curves used to calculate concentrations of individual cytokines are established by fitting the calibrator signals to a four-parameter logistic model with a 1/Y^2^ weighting. The MSD analysis software determines individual cytokine concentrations from electrochemiluminescent signals via backfitting to the calibration curve. Calculated concentrations are also multiplied by the dilution factor applied to the samples, which in this case, was 4. Samples were excluded if %CV was higher than 25% between duplicates as previously described (Dabitao et al., 2011). Lower and upper limits of detection (LLOD and ULOD) as well as interassay coefficients of variation (CVs) for each protein are outlined in Table 1. Limits of detection represent calculated concentrations which correspond to signals 2.5 standard deviations above/below the blank (zero calibrator).

**Table 1:** Median LLOD and ULOD for each of the tested cytokines

Proteins	Median LLOD (pg/ml)	Median ULOD (pg/ml)	Interassay CV (%)
IL-17A	1.60	6560.00	8.63
IFN-γ	0.34	1400.00	10.13
Eotaxin	0.44	1820.00	12.04
MCP-1	0.13	530.00	10.43
IL-16	0.83	3400.00	6.35
IL-1β	0.14	575.00	9.07
IL-6	0.19	765.00	8.76
IL-8	0.15	599.00	9.18

All units are pg/ml

Samples were chosen due to early ASD presentation (formal diagnosis prior to 5 years) and sample availability. Several were excluded from the individual final analyses due to either poor %CV values or concentrations reading below the LLOD for individual cytokines. Of the combined 25 cases and 38 controls, the final sample numbers for cases after all exclusions are outlined in Table 2.

**Table 2:** Final sample numbers for combined Cork and Auckland cases and controls

Proteins	Cases 15 weeks	Controls 15 weeks	Excluded 15 weeks	Cases 20 weeks	Controls 20 weeks	Excluded 20 weeks	Total excluded (below LLOD)	Total excluded (%CV > 25%)
IL-17A	20	34	9	20	31	12	10	11
IFN-γ	20	28	15	19	30	14	2	27
Eotaxin	15	23	25	18	16	29	7	47
MCP-1	21	32	10	19	32	12	1	22
IL-16	22	35	6	21	37	5	5	6
IL-1β	14	19	30	14	22	27	25	32
IL-6	20	28	15	20	29	14	1	28
IL-8	22	28	13	19	29	15	0	28

Derived from the original 25 cases and 38 controls

### Statistical Analysis

Statistical analysis was performed using GraphPad Prism 7 (Graphpad Software Inc., San Diego, CA) and IBM SPSS Statistics 24/26 (SPSS Statistics, Chicago, IL). All cytokine variables were log_10_ transformed prior to analysis to achieve normality (Feng et al., 2014). Independent samples t-tests were used to investigate differences between cases and controls for the cytokine variables. Multiple logistic regression models were used to assess whether cytokine concentrations can predict ASD outcome after adjusting for individual confounding variables. A confounder was defined as a variable that was associated with both case/control status and the cytokine variable under investigation. For comparisons of continuous variables between groups, independent samples t-tests were used when there were two groups and one-way ANOVAs were used when there were more than two groups. Relationships between categorical variables were investigated using the chi-squared test. Statistical significance (2-tailed) was set at p ≤ 0.05 and all tests were two-sided.

## Results

### Participant Details

Of the 2034 mothers recruited to SCOPE-NZ, 1208 agreed to participate in the follow up birth cohort study, Children of SCOPE. 16 NZ children who completed developmental follow-up and had an ASD diagnosis by 6 years were selected for cytokine profiling (compared to 16 controls). While the NZ cohort was originally matched, one case was excluded from analysis due to possible chromosomal abnormality, and its corresponding control was one of only two females remaining in the cohort, so was not excluded, resulting in 15 NZ cases total.

Of the 2183 mothers recruited to Cork’s Baseline birth cohort study, 1537 were recruited from SCOPE Ireland at the 20 weeks visit and an additional 600 children were recruited to the cohort postnatally. In total, 1249 children completed 5 year follow up assessment in the Cork BASELINE Birth Cohort Study. Of these children, 23 had a reported diagnosis of ASD, and 10 had available mid-gestation samples and were selected for cytokine profiling (compared to 22 controls). Cases selected from the Cork cohort were contacted via telephone by the study clinical research fellow in June/July 2019, and all cases were verbally confirmed to have ASD (diagnosed by local EIS or child psychologist). While the Cork cohort was originally matched, numerous samples were excluded, resulting in a lack of matching.

The cohort of ASD cases from NZ and Cork were combined (n = 25), and samples from the mothers of these children were analysed alongside those from the mothers of neurotypical controls n = 38.

Detailed clinical characteristics of participants and mothers from both cohorts are provided in Table 3. As previously stated, several samples from both locations were excluded from the final analysis due to either poor %CV values or concentrations reading below the LLOD for individual cytokines. This resulted in an altered male/female ratio between cases and controls and ultimately an unmatched cohort. Other significant differences between cases and controls included mode of delivery and folate use in early pregnancy (15 weeks).

**Table 3:** Demographic characteristics of participants

Demographics for combined NZ and IRE cohorts (n = 63)			
Variables	Cases (n = 25)	Controls (n = 38)	p-value
Age (maternal), years	30.4 (5.7)	30.6 (3.6)	0.9
Birthweight, g	3604.0 (666.0)	3439.0 (431.0)	0.2
Sex (infant)			0.02
Male	23 (92)	25 (66)	
Female	2 (8)	13 (34)	
Mode of delivery			0.04
Unassisted vaginal	9 (36)	16 (42)	
Assisted vaginal	4 (16)	15 (40)	
Pre-labour LSCS	1 (4)	2 (5)	
Labour LSCS	11 (44)	5 (13)	
Gestational age at delivery	39.9 (1.5)	40.0 (1.4)	0.9
1-min Apgar			0.08
< 7	2 (8)	0	
≥ 7	23 (92)	38 (100)	
5-min Apgar			*
< 7	0	0	
≥ 7	25 (100)	38 (100)	
Ethnicity			1
Caucasian	23 (92)	35 (92)	
Non-Caucasian	2 (8)	3 (8)	
SEI (maternal)	52.6 (16.2)	49.8 (11.7)	0.4
Household income			0.4
Unknown	2 (8)	2 (5)	
< $75 K (< €64 K)	6 (24)	11 (29)	
$75–100 K (€64–84 K)	10 (40)	8 (21)	
> $100 K (> €85 K)	7 (28)	17 (45)	
Smoking status in pregnancy			0.4
No, never smoked	20 (80)	24 (63)	
No, ex-smoker	4 (16)	11 (29)	
Yes, current smoker	1 (4)	3 (8)	
PSS (perceived stress score)	13.8 (7.3)	14.7 (6.7)	0.6
BMI (WHO categories)			0.2
Underweight/Normal (≤ 25 kg/m^2^)	14 (56)	27 (71)	
Overweight/Obese (> 25 kg/m^2^)	11 (44)	11 (29)	
Folate—pre-conceptual			0.6
No	9 (36)	11 (29)	
Yes	16 (64)	27 (71)	
Folate—15 week visit			0.02
No	3 (12)	15 (40)	
Yes	22 (88)	23 (61)	

Comparison is made between cases and controls across the whole cohort. p-Values are calculated using the Pearson Chi square for categorical data, and independent samples t-test where appropriate for continuous variables. Variations in local Caesarean section practices from each site likely give rise to the significant difference in Mode of Delivery rates. Eight of eleven (73%) of the ASD cases delivered by lower segment Caesarean section—“Labour LSCS” were in NZ. “Pre-labour LSCS” was excluded when identifying confounding variables due to small sample numbers (n = 3). There are no significant differences in birth weight, either between cases and controls, or between subjects from each site. Numbers are presented as mean (SD) or n (%)

### Mid-Gestational Cytokine Analysis

To determine whether there was any difference in inflammatory markers between mothers of ASD and neurotypical children at either 15 or 20 weeks gestation, electrochemiluminescent Mesoscale assays were performed.

**Fig. 2:**
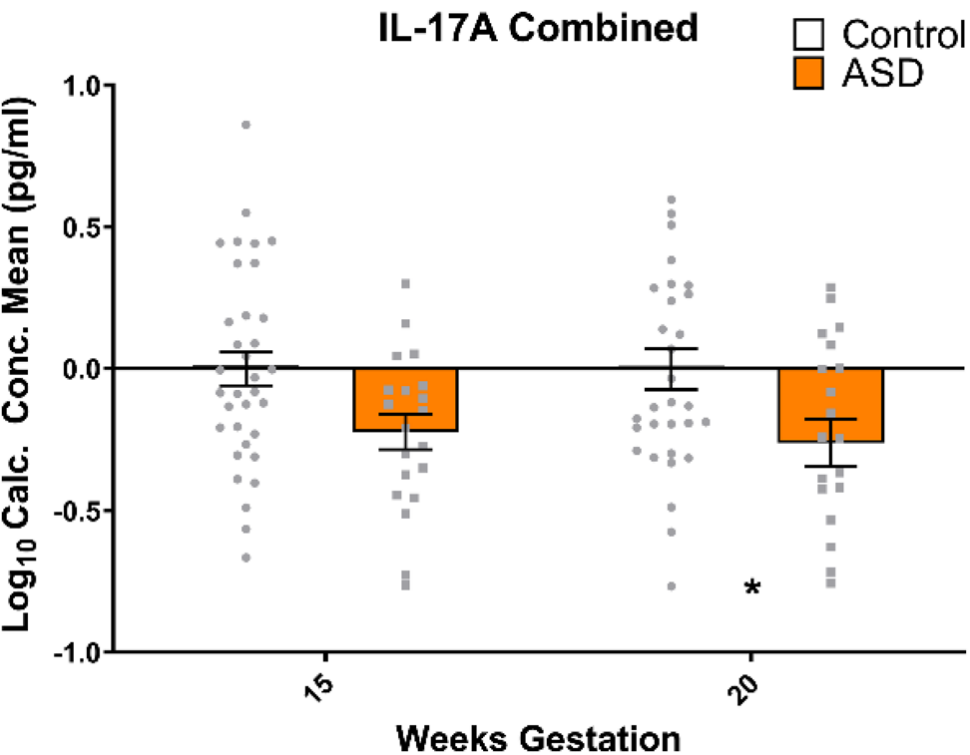
IL-17A is downregulated, at 20 weeks gestation in mothers of ASD children when compared to neurotypical controls. This remains after adjusting for confounding variables—folate intake at 15 weeks. All data are mean ± SEM; independent samples t-tests, analysed on a case vs control basis. * = p < 0.05. White bars represent controls, while orange bars represent cases (mothers of ASD affected offspring)

Of the original panel of eight cytokines, one was significantly altered—IL-17A. IL-17A was significantly altered at both 15 and 20 weeks in mothers of children who went on to have a child affected by ASD, compared to controls. IL-17A concentrations were significantly different between cases (Mean (M) = − 0.22; Standard Deviation (SD) = 0.28) and controls (M = − 0.001; SD = 0.35) at 15 weeks (t(52) = 2.43; p = 0.02), and between cases (M = − 0.26; SD = 0.38) and controls (M = − 0.002; SD = 0.40) at 20 weeks (t(49) = 2.32; p = 0.02) (Fig. 2). After adjusting for confounding by folate, IL-17A no longer showed a statistically significant association with ASD risk at 15 weeks (adjusted odds ratio [aOR] 0.17 (95% CI 0.02–1.57); p = 0.12). Downregulation at 20 weeks remained, as there were no changes in associations after adjustment for confounding by folate (aOR 0.14 (95% CI 0.02–0.87); p = 0.03).

Expression of IFN-γ, IL-16, Eotaxin, MCP-1, IL-1β, IL-8 and IL-6 was not significantly different in mothers who went on to have a child with ASD when compared to controls at either timepoint. Therefore, levels of these cytokines were not associated with increased ASD risk.

**Fig. 3:**
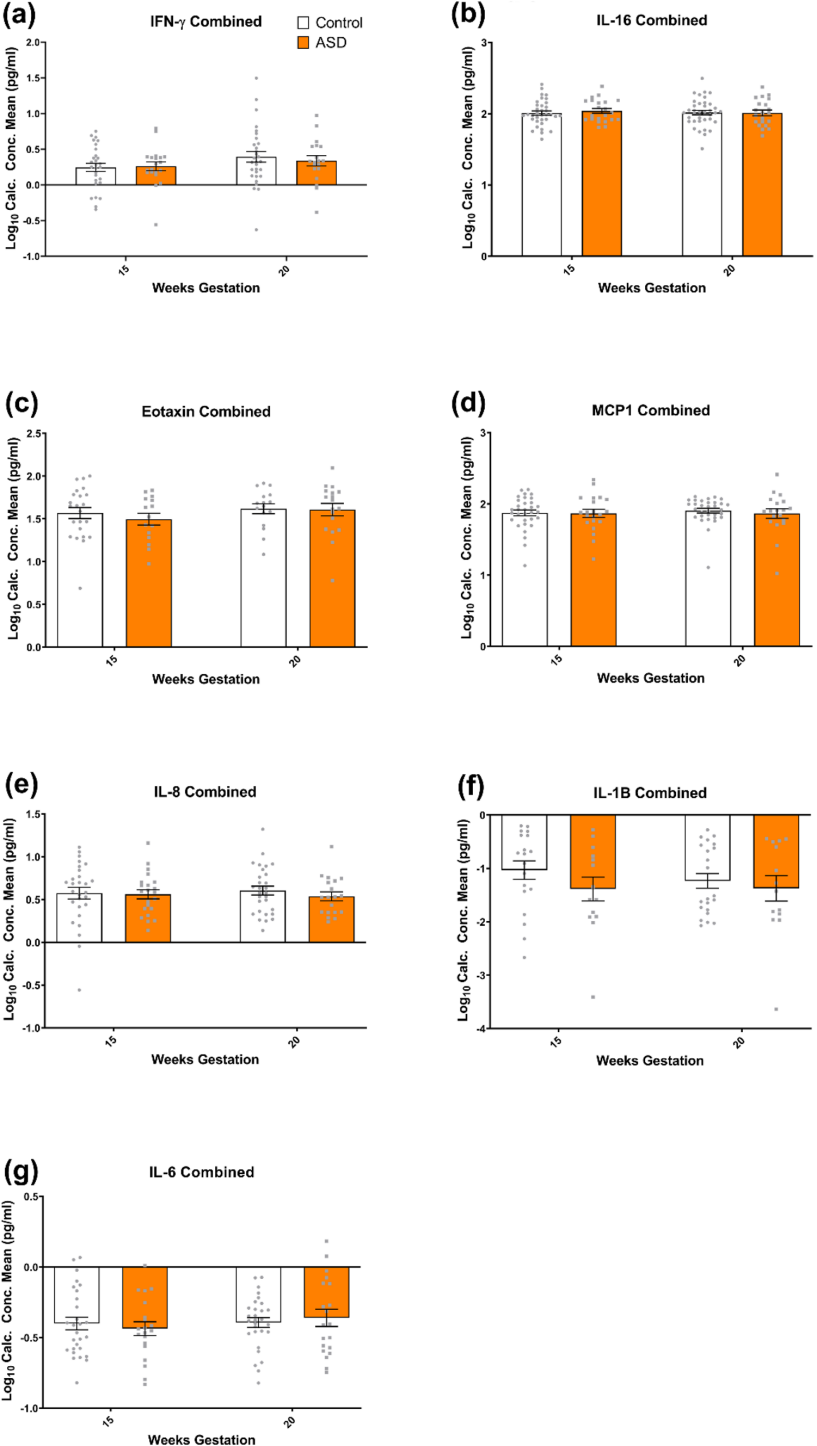
**a** IFN-γ, **b** IL-16, **c** eotaxin, **d** MCP1, **e** IL-8, **f** IL-1β and **g** IL-6 were not significantly altered at either 15 or 20 weeks gestation in mothers of ASD children when compared to neurotypical controls. All data are mean ± SEM; independent samples t-tests, analysed on a case vs control basis. White bars represent controls, while orange bars represent cases (mothers of ASD affected offspring)

IFN-γ was not found to be significantly different between cases (M = 0.26; SD = 0.28) and controls (M = 0.25; SD = 0.31) at 15 weeks (t(46) = 0.19; p = 0.85) or between cases (M = 0.34; SD = 0.31) and controls (M = 0.39; SD = 0.41) at 20 weeks (t(47) = 0.51; p = 0.62) (Fig. 3a). IL-16 was not significantly different between cases (M = 2.04; SD = 0.16) and controls (M = 2.01; SD = 0.18) at 15 weeks (t(55) = 0.64; p = 0.52), or between cases (M = 2.01; SD = 0.19) and controls (M = 2.02; SD = 0.20) at 20 weeks (t(56) = 0.12; p = 0.92) (Fig. 3b). Sex was found to be a confounder for IL-16 at 15 weeks, though levels remained not significantly associated with development of ASD after adjusting for confounding by sex (aOR 2.38 (95% CI 0.63–89.61); p = 0.64). Eotaxin was not significantly different between cases (M = 1.50; SD = 0.27) and controls (M = 1.57; SD = 0.31) at 15 weeks (t(36) = 0.73; p = 0.47), or between cases (M = 1.61; SD = 0.31) and controls (M = 1.62; SD = 0.23) at 20 weeks (t(32) = 0.11; p = 0.91) (Fig. 3c). MCP-1 was not significantly different between cases (M = 1.87; SD = 0.26) and controls (M = 1.87; SD = 0.23) at 15 weeks (t(51) = 0.10; p = 0.92), or between cases (M = 1.87; SD = 0.29) and controls (M = 1.90; SD = 0.19) at 20 weeks (t(49) = 0.58; p = 0.56) (Fig. 3d). IL-8 was not significantly different between cases (M = 0.56; SD = 0.25) and controls (M = 0.57; SD = 0.36) at 15 weeks (t(48) = 0.15; p = 0.88), or between cases (M = 0.54; SD = 0.23) and controls (M = 0.61; SD = 0.28) at 20 weeks (t(46) = 0.89; p = 0.38) (Fig. 3e). IL-1β was not significantly different between cases (M = − 1.39; SD = 0.83) and controls (M = − 1.03; SD = .75) at 15 weeks (t(31) = 1.28; p = 0.21), or between cases (M = − 1.37; SD = 0.89) and controls (M = − 1.23; SD = 0.65) at 20 weeks (t(34) = 0.54; p = 0.59) (Fig. 3f). Mode of delivery was found to be a confounder for IL-1β at 15 and 20 weeks, though IL-1β at 15 weeks (aOR 0.83 (95% CI 0.28–2.45); p = 0.74) and 20 weeks (aOR 0.76 (95% CI 0.26–2.23); p = 0.61) remained not significantly associated with development of ASD after adjusting for confounding by mode of delivery. IL-6 was not significantly different between cases (M = − 0.44; SD = 0.22) and controls (M = − 0.40; SD = 0.24) at 15 weeks (t(46) = 0.54; p = 0.59), or between cases (M = − 0.36; SD = 0.27) and controls (M = − 0.39; SD = 0.19) at 20 weeks (t(47) = 0.50; p = 0.62) (Fig. 3g). Sex was found to be a confounder for IL-6 at 15 weeks, though IL-6 at 15 weeks remained not significantly associated with development of ASD after adjusting for confounding by sex (aOR 0.30 (95% CI 0.17–5.17); p = 0.41).

### Maternal Health

To examine whether other factors might have altered maternal cytokine profiles we examined maternal health factors and medication use during pregnancy.

None of the participants had any of the following pre-existing inflammatory conditions—inflammatory bowel disease, rheumatoid or inflammatory arthritis, venous thromboembolic disease. No subjects suffered from either psoriasis or ankylosing spondylitis. The commonest reported medical condition was asthma. Several participants from each site had physician diagnosed asthma; 12 suffered from mild asthma and 3 suffered from moderate asthma. The individuals with moderately severe asthma (one case (Cork), two controls (NZ)) were being treated with regular low dose inhaled corticosteroid and long acting beta agonist or Terbutaline combination therapy. The subjects with mild asthma were 7 controls (4 Cork, 3 NZ) and 5 cases (2 Cork, 3 NZ). None of the asthmatics recieved oral steroid therapy at any point during pregnancy or in the preceeding year. Hypothyroidism was also relatively common and occurred in three cases (2 NZ, 1 Cork) and two controls (1 NZ, 1 Cork). Two of the three cases had evidence of hypothyroidism first detected during the 1st trimester and became euthyroid with treatment. Those others with a history of hypothyroidism were treated prior to pregnancy and were euthyroid throughout. Finally, a single case in Cork had Coeliac disease (on gluten free diet).

With regard to antepartum infections, between 0 and 15 weeks gestation, upper respiratory tract infections were reported in 12 subjects, 5 (4 NZ, 1 Cork) cases and 7 (5 NZ, 2 Cork) controls. Other infections were also reported in three (all NZ) cases and seven (3 NZ, 4 Cork) controls (5 gastroenteritis, 3 lower urinary tract infections (UTI), 1 case of genital herpes and another vaginal candida, treated with Clotrimazole. At 15–20 weeks no infections were reported in the NZ group, but two controls from Cork had a UTI and one case had an unspecified infection. None of the participants were taking regular anti-inflammatories and those taking paracetamol or aspirin did so only occasionally or for a specific episode. There was no significant difference between case and control groups in terms of reported paracetamol/aspirin use.

In summary, there were no significant differences in maternal health, inflammation or medication use between the two groups.

## Discussion

In the present report, we have identified IL-17A as a potential cytokine biomarker whose expression is significantly reduced in mid-gestation (20 weeks) in pregnancies resulting in a child with ASD after adjusting for folate intake at 15 weeks. This novel finding adds to the growing body of evidence that in utero exposure to MIA and resultant cytokine dysfunction is associated with an increased risk of the subsequent development of ASD.

Interestingly, the potential confounders identified within this study—sex, mode of delivery and maternal folate intake—are widely discussed risk factors for the development of ASD (Curran et al., 2015a, 2015b; Gillberg et al., 2006; Raghavan et al., 2018; Wiens & DeSoto, 2017). After adjusting for maternal folate intake at midgestation, IL-17A levels at 15 weeks were no longer significantly associated with ASD development in offspring. A high number of case subjects (22) answered ‘yes’ to taking folate supplements during midgestation, while only three answered no. While no data are available on the doses of folate taken here, studies have linked both low and high dose maternal folate intake to DNA hyper/hypomethylation, gamma-aminobutyric acid (GABA), dopamine and serotonin dysfunction, and altered synaptic plasticity, neurogenesis and growth cone development. These events trigger neurodevelopmental disturbances which may lead to the development of ASD (DeVilbiss et al., 2015; Raghavan et al., 2018; Wiens & DeSoto, 2017). As previously mentioned, the current study had a larger ratio of males to females. It is widely understood that ASD is more commonly diagnosed in males. There are a number of theories on why this is the case. It appears that males may tend to externalise symptoms of the disorder, whereas females typically internalise symptoms, complicating diagnosis for females (Baron-Cohen et al., 2011; Werling & Geschwind, 2013). Mode of delivery was also identified as a confounder. Indeed, over 50% of mothers of ASD cases delivered by C-section which was initiated after the onset of labour. Emergency C-section is typically preceded by either foetal or maternal indications which may themselves be independent risk factors for ASD (Yip et al., 2017). C-section delivery has been linked to impaired cognitive and behavioural outcomes in both humans and animal models (Curran et al., 2015a, 2015b; Morais et al., 2020; Polidano et al., 2017). Delivery by C-section has been linked to reductions in endogenous oxytocin (Kuwabara et al., 1987), and subsequent social deficits in mice. These deficits may be reversed in mice by exogenous oxytocin therapy early during the postnatal period (Morais et al., 2021).

Although this is one of the few human studies to examine maternal midgestation cytokine dysregulation linked to ASD, there is an abundance of data from animal studies on the cytokine and behavioural changes resulting from MIA. MIA has been replicated in small animal models where induction of MIA through maternal infection leads to an autistic phenotype in offspring, characterised in mice by increased self-grooming, increased marble burying behaviour (repetitive, stereotyped behaviours) and deficits in ultrasonic vocalisations (communication). These alterations maybe prevented by inhibition of specific cytokines (IL-6 and IL-17A), which suggests that the cytokines themselves may have a causative role in the resultant neuronal dysfunction (Parker-Athill & Tan, 2010; Smith et al., 2007; Wong & Hoeffer, 2018).

In the murine MIA model of ASD, Poly(I:C) treatment has been found to increase IL-17A levels in maternal blood and the postnatal brain as well as placental messenger RNA (mRNA) levels of the cytokine. To determine whether alterations in IL-17A expression are symptomatic of, or pathogenic in ASD, a recent study inhibited IL-17A signalling in Poly(I:C) treated pregnant mice and reported that ASD-like phenotypes in the offspring were prevented (Choi et al., 2016). IL-17A and IL-6 appear to work in tandem. Knockout of IL-6 in Poly(I:C) treated dams results in failure to alter IL-17A levels in offspring, which suggests IL-6 acts upstream of IL-17A (Choi et al., 2016). Poly(I:C) is a synthetic analogue of double stranded RNA which mimics the effects of viral infection when injected into test subjects (Meyer & Feldon, 2012). It is used as a model of MIA extensively in rat, mouse and non-human primate studies. Pups of MIA-exposed dams in Poly(I:C) murine models have demonstrated communication challenges, reduced social approach, increased repetitive behaviours (Choi et al., 2016) and alterations in developement of the cerebral cortex and cerebellum (Garay et al., 2013; Hsiao et al., 2012).

Accumulating evidence supports a role for T-helper 17 (Th17) cluster of differentiation 4 (CD4) cells and their product cytokine IL-17A in ASD. Th17 cells have previously been implicated in the pathogenesis of a variety of autoimmune and neuroinflammatory disorders (Al-Ayadhi & Mostafa, 2012). Upstream IL-6 is also a key player in differentiation of these Th17 cells (Choi et al., 2016). Th17/IL-17 mediated immunity has been found to cause severe damage to the brain in response to inflammation-sensitised hypoxia (Yang et al., 2014). The gene for IL-17A (IL17A) has been identified in a genome-wide analysis to have enriched/overexpressed copy number variants in ASD cohorts (van der Zwaag et al., 2009). In subsets of children with ASD, IL-17A has been found at elevated levels in the blood (both plasma and serum) and correlated with increased severity of behavioural symptoms (Akintunde et al., 2015; Al-Ayadhi & Mostafa, 2012). Nadeem et al. report that children affected by ASD have an increased number of IL-17A receptors in monocytes and that activation via IL-17A increases the child’s oxidative inflammation. Blocking the receptor may ameliorate inflammatory effects, which suggests an interesting therapeutic option for both inflammatory and behavioural symptoms (Nadeem et al., 2018). Indeed, IL-17A administration in a murine model improves sociability following MIA (Reed et al., 2020). IL-17A/IL-17A receptor blockade has also been shown to ameliorate the symptoms of other disorders such as atherosclerosis (Erbel et al., 2009), inflammation-sensitised encephalopathy (Ye et al., 2019) and anklyosing spondylitis (Collison, 2018). IL-6 has been detected at elevated levels in cerebellar tissues of humans affected by ASD in their lifetime. Altered levels of this cytokine have been linked to dysfunctional adhesion and migration of neural cells, as well as imbalanced excitatory and inhibitory functions. This suggests that altered expression of IL-6 may contribute to the autistic phenotype and pathogenesis (Wei et al., 2011). Levels are also significantly increased in the frontal cortex and plasma of ASD patients (Li et al., 2009; Yang et al., 2015). Elevated IL-6 in the murine brain also results in an autistic behavioural phenotype, as well as abnormal dendritic morphology and distribution (Wei et al., 2012). Though we do not observe any notable alterations in IL-6 in this study, perhaps it acts at later timepoints when the nervous system is more developmentally mature.

The present study has a number of strengths which increase our confidence in the findings. It involves a multi-centre, multi-national maternal cohort of over 4000 women, with very detailed maternal demography and 1st trimester health and lifestyle data at 15 weeks gestation. Of these women, 39 went on to have a child affected by ASD (~ 1% ASD rate). The rate of ASD seen in this cohort is similar to that seen across the developed world (~ 1.5%), so this study is a realistic reflection of ASD incidence. For this reason, we are confident that we have identified the majority of expected cases. Serum samples from both SCOPE study centres were collected, processed and biobanked according to identical protocols to ensure uniformity. Though it appears that our finding IL-17A downregulation goes against the previous reports regarding induced upregulation of IL-17 in animal studies (Wong & Hoeffer, 2018), one must consider that this is currently one of the only studies in humans which has examined IL-17A in midgestation, and is therefore a novel finding. There is increasing evidence that IL-17A may cross the placenta from mother to foetus (Wong & Hoeffer, 2018), which may, in theory, explain reduced levels in maternal serum and increased levels typically seen in the serum of offspring.

Though the present study has some major strengths, we must also address its limitations. One major shortcoming of the current study is its inability to replicate the findings of similar mid-gestation ASD cytokine studies (Abdallah et al., 2012; Goines et al., 2011; Jones et al., 2017). However, results are conflicting amongst the previous studies. Goines et al. reported midgestational elevation of IFN-γ in mothers of children who develop ASD, which contrasts with the current study (Goines et al., 2011). Jones et al. from the same research group detected midgestational downregulation of IL-8 and MCP-1 in mothers of children who develop ASD without intellectual disability. We did not find significant alterations in these cytokines in our cohort (Jones et al., 2017). Abdallah et al. utilised amniotic fluid to profile elevated MCP-1 in mothers of children who developed ASD. While we see very slight downregulation of MCP-1 at 20 weeks, Abdallah et al. do not specify weeks gestation at measurement in their study (Abdallah et al., 2012).The differences in findings between studies may relate to several factors: assays and measurement of cytokines (Luminex/Millipore—neither used Mesoscale assays), differences in the stage of gestation at measurement, and our small study size compared to other similar studies.The relatively small numbers of ASD cases makes it difficult to draw meaningful conclusions regarding different sub-types of ASD. A number of ASD samples were also lost due to poor quality, reflected by large but inconsistent (across multiplex plates) numbers lost due to poor %CV, further reducing our cohort size, subsequentially resulting in a disproportionately large percentage of male cases compared to females. In addition to this, a large number of samples were below the LLOD for many cytokines (up to 25—Table 2), which suggests the assay used may not have been sensitive enough. This ultimately created an unmatched cohort. The follow-up procedure was different at both sites, with a more detailed follow up at 2 and 5 years available to the Cork BASELINE study. However, the diagnosis of ASD was similar: parental report (Auckland), parental report of confirmed EIS or psychiatrist diagnosis. In Cork, children were diagnosed relatively early and so may be more on the severe end of the spectrum to that in Auckland. A large percentage of cases were delivered via Caesarean section. This may skew results as this mode of delivery has previously been associated with increased ASD incidence (Al-Zalabani et al., 2019; Curran et al., 2015a, 2015b; Morais et al., 2020). Larger, longer-term studies, which take long-term outcomes into account will be required with repeated maternal cytokine profiling to attempt to replicate and expand our findings.

## Conclusion

To conclude, this study has identified dysfunctional IL-17A expression at 20 weeks gestation in mothers of ASD children. IL-17A may act as a potential early marker of maternal immune dysfunction and if validated would aid screening of high risk infants to support focused early therapeutic intervention in infancy (Josefi & Ryan, 2004). The current study provides a foundation for further investigation of IL-17A in large maternal cohorts. This multicentre study also provides novel insight into the midgestation cytokine profiles in mothers of both neurotypical and ASD offspring and is another piece in the puzzle of this elusive disorder.
